# Splanchnic venous thrombosis in a nephrotic patient following COVID-19 infection: a case report

**DOI:** 10.1186/s12882-021-02643-0

**Published:** 2021-12-29

**Authors:** Maged H. Hussein, Mohamad S. Alabdaljabar, Noorah Alfagyh, Mohammad Badran, Khalid Alamiri

**Affiliations:** 1 Department of Medicine, King Faisal Specialist Hospital and Research Center, MBC-46, P.O.Box 3354, Riyadh, 11211 Saudi Arabia; 2 College of Medicine, Alfaisal University, Riyadh, Kingdom of Saudi Arabia; 3 Department of Radiology, King Faisal Specialist Hospital and Research Center, Riyadh, Kingdom of Saudi Arabia

**Keywords:** SARS-CoV-2, Nephrotic syndrome, Splanchnic vein thrombosis, Portal vein thrombosis, Hypercoagulable state

## Abstract

**Background:**

As the COVID-19 pandemic spread worldwide, case reports and small series identified its association with an increasing number of medical conditions including a propensity for thrombotic complications. And since the nephrotic syndrome is also a thrombophilic state, its co-occurrence with the SARS-CoV-2 infection is likely to be associated with an even higher risk of thrombosis, particularly in the presence of known or unknown additional risk factors. Lower extremity deep vein thrombosis (DVT) and pulmonary embolism (PE) are the most common manifestations of COVID-19-associated hypercoagulable state with other venous or arterial sites being much less frequently involved. Although splanchnic vein thrombosis (SVT) has been reported to be 25 times less common than usual site venous thromboembolism (VTE) and rarely occurs in nephrotic patients, it can have catastrophic consequences. A small number of SVT cases have been reported in COVID-19 infected patients in spite of their number exceeding 180 million worldwide.

**Case presentation:**

An unvaccinated young adult male with steroid-dependent nephrotic syndrome (SDNS) who was in a complete nephrotic remission relapsed following contracting SARS-CoV-2 infection and developed abdominal pain and diarrhea. Abdominal US revealed portal vein thrombosis. The patient was anticoagulated, yet the SVT rapidly propagated to involve the spleno-mesenteric, intrahepatic and the right hepatic veins. In spite of mechanical thrombectomy, thrombolytics and anticoagulation, he developed mesenteric ischemia which progressed to gangrene leading to bowel resection and a complicated hospital course.

**Conclusion:**

Our case highlights the potential for a catastrophic outcome when COVID-19 infection occurs in those with a concomitant hypercoagulable state and reminds us of the need for a careful assessment of abdominal symptoms in SARS-CoV-2 infected patients.

## Background

The nephrotic syndrome as well as COVID-19 infection are associated with an increased risk of thrombosis. Splanchnic vein thrombosis (SVT) which involves one or more of the portal, splenic, superior mesenteric or hepatic veins is much less common than usual sites venous thromboembolism (VTE) [1], and has rarely been reported in nephrotic patients [2]. It has also been reported in COVID-19 infected patients [3, 4] and following vaccination with the adenoviral vector-based ChAdOx1 nCoV-19 (AstraZeneca) [5]. Herein, we report an unvaccinated, young adult male with steroid-dependent nephrotic syndrome (SDNS) who relapsed following SARS-CoV-2 infection and developed portal vein thrombosis (PVT) which rapidly propagated to the spleno-mesenteric, intrahepatic and the right hepatic veins resulting in intestinal gangrene.

## Case presentation

A twenty-year-old college student was seen for a relapse of his nephrotic syndrome. He was in his usual state of health until 3 weeks earlier when he developed anosmia and ageusia and tested positive for COVID-19. He had no other respiratory symptoms but the following week, he developed anasarca, diarrhea and abdominal pain.

His medical history was remarkable for steroid-dependent nephrotic syndrome, morbid obesity, hypertension and pulmonary embolism (PE). His nephrosis started at the age of 4 years, relapsed about twice yearly and responded to corticosteroids. A kidney biopsy at age 18 years revealed minimal change disease (MCD) histology. He suffered a sub-massive PE while in relapse 4 months later. That nephrotic relapse was treated initially with oral prednisone followed by rituximab. Glucocorticoids (GC) were tapered and discontinued. In his most recent clinic follow-up, he had been off both GC and anticoagulants, in complete nephrotic remission and with normal renal function.

**Fig. 1:**
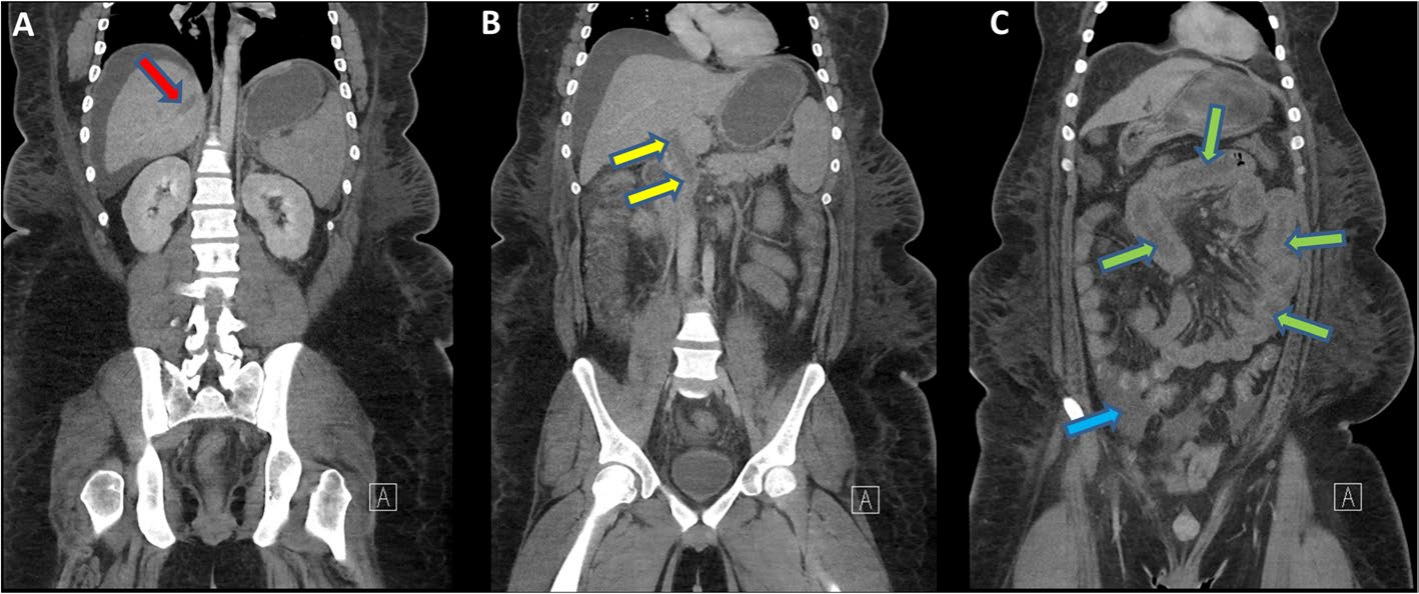
Coronal CT reconstruction showing thrombosed right hepatic (red arrow) and portal veins (yellow arrows), congested small bowel (green arrows) and small ascites (blue arrow)

On admission, weight was 153 kg with a BMI of 48.1 kg/m^2^ and blood pressure was 83/50. Physical exam was unremarkable except for anasarca. Labs showed an elevated serum creatinine level, hypoalbuminemia and proteinuria (Table 1). He was given intravenous fluids with improvement in blood pressure and a decrease in serum creatinine. Oral prednisone was initiated at 60 mg daily together with subcutaneous unfractionated heparin thromboprophylaxis. Abdominal ultrasound showed PVT. Therapeutic dose intravenous heparin was started and the patient was discharged on oral prednisone and rivaroxaban 20 mg daily. He returned the next day with severe abdominal pain and hematochezia. Labs showed increase in WBC count to 23.0 × 10 [6]/l, AST to 723 IU/l and ALT 708 IU/l. Chest x-ray was unremarkable. CT scan showed portal, splenic, mesenteric and right hepatic veins thrombosis with bowel congestion and small ascites but no signs of advanced intestinal ischemia (Fig. 1). Lactate level was 2 mmol/l.

**Table 1:** Laboratory results of our patient on admission

Serum	
Creatinine (μmol/l)	289 H (64–115)
BUN (mmol/l)	15.6 H (2–6.2)
Albumin (g/l)	17 L (40–50)
Hemoglobin (g/l)	153 (135–180)
MCV (fl)	86.9 (75–95)
WBC (10^9^/l)	11.2 H (3.9–11.0)
Platelets (10^9^/l)	361 (155–435)
PT (s)	16.2 H (12.3–14.2)
INR	1.2 H (0.9–1.1)
PTT (s)	39 (30.5–40.4)
AST (IU/l)	32.6 (10–45)
ALT (IU/l)	41.3 (10–45)
Bilirubin, total (mmol/l)	3 (0–21)
Lactate (mmol/l)	1.1 (0.05–2.0)
COVID-19 PCR	Negative
Urine	
Protein (g/l)	7.05 H (0–0.14)
Creatinine (mmol/l)	75 H (1.8–28.3)
P/C Ratio (mg/mmol)	95.27 H
pH	5.0
Specific gravity	> 1.030 H
Protein	3+ H
Blood	Negative
Bilirubin	3+ H

*P/C* Protein/creatinine

**Fig. 2:**
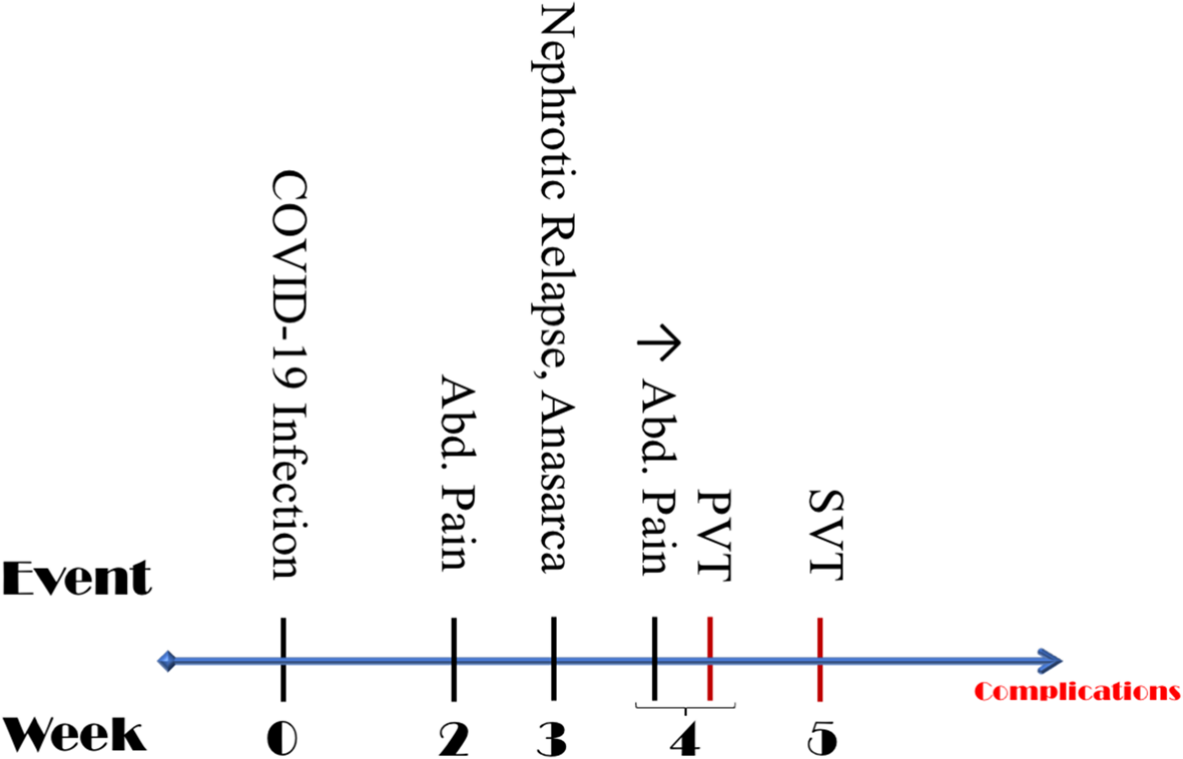
Timeline of events. *Abd.*: Abdominal; *PVT*: Portal vein thrombosis; *SVT*: Splanchnic vein thrombosis (portal, splenic, mesenteric and right hepatic veins)

Catheter directed thrombectomy and thrombolysis were performed and a transjugular intrahepatic portosystemic shunt was inserted after recanalization of the right hepatic vein. That was followed by systemic thrombolysis and heparin anticoagulation. Fibrinogen level increased from 5.5 g/l following admission to 7.84 g/l over 4 days and remained elevated for the following week. CRP was 30.3 and increasing over 2 days to > 300 mg/l. Sepsis ensued and was complicated by a second episode of acute kidney injury (AKI) requiring renal replacement therapy (RRT), acute respiratory failure, severe fluid overload and abdominal compartment syndrome. Abdominal CT revealed intestinal infarction with pneumatosis intestinalis and pneumoperitoneum necessitating an emergency laparotomy with extensive resection of the necrotic bowel. His renal function fully recovered, and by hospital week 9, a complete nephrotic remission was achieved with continued GC therapy. After a long, complicated hospital course, he was discharged home with short bowel syndrome on lifelong anticoagulation (Fig. 2). On last follow-up 11 months after presentation, his bowel habits have normalized, he remains in complete nephrotic remission and his oral prednisone was discontinued. He also received 2 doses of rituximab.

Workup for additional hypercoagulable risk factors was negative for lupus anticoagulant, antibodies to cardiolipin, phosphatidylserine and B2-glycoprotein. He also tested negative for factor V Leiden, MTHFR, JAK2 v617f and prothrombin G 20210 mutations. Subsequent testing at week 9 showed normal functional protein C and antithrombin III levels. Functional protein S level measured on 2 occasions (9 and 18 weeks) was mildly decreased at 0.56 and 0.46 IU/ml respectively (N 0.6–1.4 IU/ml), both with normal free protein S levels, suggestive of a mild type II deficiency. Test for activated protein C (APC) resistance was negative. Flow-cytometry was negative for paroxysmal nocturnal hemoglobinuria (PNH). Homocysteine level was mildly elevated at 23 μmol/l, which then normalized. His COVID-19 PCR was intermittently positive for 3 weeks after his initial presentation while his SARS-CoV-2 total, IgM and IgG antibodies were positive.

## Discussion

In addition to the elderly, the SARS-CoV-2 pandemic has disproportionately impacted younger patients with comorbid conditions [7]. As it unfolded, a marked increase in the risk of venous and arterial thrombosis in those hospitalized with the infection became evident. This was attributed to a combination of factors including hypoxemia, inflammation, endothelial injury, hyperfibrinogenemia, elevated levels of VW factor as well as the presence of neutrophil extracellular traps (NETs) [8]. Published guidelines recommend anticoagulant thromboprophylaxis for hospitalized COVID-19 patients [9]. For outpatients, however, anticoagulants are only considered in those with additional VTE risk factors [6].

Nephrotic patients lose natural anticoagulants such as antithrombin-III, proteins C and S in their urine. Hyperfibrinogenemia, plasminogen activator inhibition, the presence of high molecular weight fibrinogen moieties and platelet activation also contribute to the prothrombotic state. Anticoagulation decisions are based on the underlying etiology, serum albumin level, bleeding risk and the presence of other risk factors [10].

SVT is an atypical site thrombosis with an incidence at least 25 times less than that usual site VTE [1]. In a prospective study including 191 nephrotic patients, MCD was found to have the least prevalence rate of VTE (4%) among nephrotic conditions [11]. COVID-19 infection has been associated with increase the risk of VTE with a recent meta-analysis estimating it to be 26% in hospitalized patients. It mainly manifested as DVT or PE [12], although numerous case reports and small series of arterial as well as unusual site venous thrombosis continue to be published. To the best of our knowledge, no studies have directly assessed the incidence/prevalence of SVT in MCD or with COVID-19 infections. VTE risk factors are many and can be classified as genetic or acquired, provoking or non-provoking and modifiable or non-modifiable. Infection is a recognized risk factor for both nephrotic relapses and VTE [8, 10, 13]. Nephrotic relapses following COVID-19 infection have been reported with and without thrombotic complications [14–19]. Our patient developed abdominal symptoms after the diagnosis of COVID-19 infection as may occur in up to a half of those infected with this virus [20]. His RT-PCR was negative when he presented, similar to the case of PVT reported by Franco-Moreno et al. [6] Upon ultrasound diagnosis of portal vein thrombosis, he was started on unfractionated heparin anticoagulation followed by rivaroxaban. Nonetheless, thrombosis soon progressed to involve the splenomesenteric and right hepatic veins. Failure to restore vascular patency with percutaneous mechanical thrombectomy, catheter-directed and systemic thrombolysis, and unfractionated heparin anticoagulation lead to intestinal gangrene. Our patient’s SVT was likely a manifestation of severe hypercoagulability brought about by a combination of obesity, the nephrotic syndrome and the recent COVID-19 infection. The mildly decreased functional protein S level which was likely even lower during each nephrotic relapse could have also added to his thrombosis risk. And while the COVID-19 infection diagnosis clearly preceded nephrotic symptoms, the exact onset of the SVT cannot be determined with certainty as abdominal symptoms are common with both conditions. Clot extension in spite of anticoagulant therapy may reflect anticoagulant failure resulting from, among other factors, his morbid obesity and severe hypoalbuminemia.

“Atypical site thrombosis” is rare and usually associated with thrombophilia [1, 2]. Multiple cases of mesenteric ischemia in COVID-19 patients have been reported. Most were arterial with only a few cases of venous ischemia [13]. Those affected mostly had average VTE background risk.

Our patient had other VTE risk factors, in addition to the nephrotic syndrome and the COVID-19 infection, namely morbid obesity, PE history and a low functional protein S level. His anticoagulation had been discontinued a year earlier since his previous PE was considered to have been provoked by the nephrotic state which subsequently resolved with treatment. The natural history of his kidney disease, however, is a relapsing one and, with it, its associated thrombosis risk. Following rituximab treatment of patients with SDNS, the median time to relapse is about 1 year [21]. The comparative efficacy of anticoagulants in the nephrotic syndrome and in COVID-19 infection is not known [8].

A recent syndrome termed vaccine-induced immune thrombotic thrombocytopenia “VITT” has been described following vaccination with some adenoviral vector-based COVID-19 vaccines [5, 22] particularly the AstraZeneca ChAdOx1nCoV-19 vaccine which uses a recombinant chimpanzee adenoviral vector encoding the spike protein. It is characterized by thrombocytopenia and unusual site thrombosis, mainly cerebral and splanchnic. Both features appear to be the result of platelet activating antibodies which interact with platelet factor 4/polyanion complexes in a manner similar to heparin-induced thrombocytopenia (HIT). This, however, has not been described in cases of COVID-19 infection associated atypical site thrombosis, the mechanism of which remains unknown. As both conditions are rare, those affected likely have additional acquired or hereditary predisposition. Heritability of VTE risk has been estimated to be 50–60%, and the majority of this risk remains unknown [23]. Genomic studies in those patients may uncover known or novel susceptibility loci.

In conclusion, we present a case of SVT following COVID-19 infection and in association with a nephrotic relapse in a morbidly obese 20-year-old male with SDNS, type 2 protein S deficiency and a previous history of pulmonary embolism. The contribution of COVID-19 infection to this serious condition could have been twofold; directly by virtue of its associated hypercoagulability and indirectly by inducing a relapse of a second prothrombotic state, namely the nephrotic syndrome (Table 2).

**Table 2:** Teaching points

1. Abdominal pain in COVID-19 patients could be a harbinger of serious pathology such as intestinal ischemia and warrants careful assessment	
2. Patients with a priori VTE risk factors are at a higher risk for thrombotic events when infected with SARS-CoV-2, even as outpatients	
3. Prothrombotic risk is dynamic requiring vigilance on the part of the health care team	
4. Past history of VTE, even if provoked, is a major risk factor for recurrence	
5. A relapse of the nephrotic syndrome should dictate immediate assessment of VTE risk and consideration of urgent anticoagulation in high-risk individuals	

*VTE* Venous thromboembolism

## Data Availability

The datasets used and/or analyzed during the current study are available from the corresponding author upon a reasonable request.

## Abbreviations

AKI: Acute kidney injury
APC: Activated protein C
GC: Glucocorticoids
HIT: Heparin-induced thrombocytopenia
MCD: Minimal change disease
PE: Pulmonary embolism
PNH: Paroxysmal nocturnal hemoglobinuria
RRT: Renal replacement therapy
SDNS: Steroid-dependent nephrotic syndrome
SVT: Splanchnic vein thrombosis
VTE: Venous thromboembolism

## References

[1] Valeriani E, Riva N, Di Nisio M, Ageno W (2019). Splanchnic vein thrombosis: current perspectives. Vasc Health Risk Manag.

[2] De Stefano V, Martinelli I (2012). Abdominal thromboses of splanchnic, renal and ovarian veins. Best Pract Res Clin Haematol.

[3] Singh B, Kaur P, Maroules M. Splanchnic vein thrombosis in COVID-19: a review of literature. Dig Liver Dis. 2020;29. 10.1016/j.dld.2020.09.025 PMC7522621 33067157

[4] Hassan AA, Alsaleh ME, Alsaleh ME, Al Zaher FA, Almajed FA, Alkhudhair AM, Alali MM, Alzayer HA, Alolayan AJ. Budd-Chiari Syndrome: A Case Report of a Rare Presentation of COVID-19. Cureus. 2021;13(1). 10.7759/cureus.12554 PMC7863030 33564546

[5] Muir KL, Kallam A, Koepsell SA, Gundabolu K. Thrombotic thrombocytopenia after Ad26. COV2. S vaccination. N Engl J Med. 2021;14. 10.1056/NEJMc2105869 PMC8063883 33852795

[6] Franco-Moreno A, Piniella-Ruiz E, Montoya-Adarraga J, Ballano-Franco C, Alvarez-Miguel F, Peinado-Martinez C, Landete-Hernandez E, Saez-Vaquero T, Ulla-Anes M, Torres-Macho J (2020). Portal vein thrombosis in a patient with COVID-19. Thromb Res.

[7] Benelli G, Buscarini E, Canetta C, La Piana G, Merli G, Scartabellati A, et al. SARS-COV-2 comorbidity network and outcome in hospitalized patients in Crema. Italy medRxiv. 2020;1. 10.1371/journal.pone.0248498 PMC7993836 33765013

[8] Singhania N, Bansal S, Nimmatoori DP, Ejaz AA, McCullough PA, Singhania G (2020). Current overview on hypercoagulability in COVID-19. Am J Cardiovasc Drugs.

[9] Cuker A, Tseng EK, Nieuwlaat R, Angchaisuksiri P, Blair C, Dane K, Davila J, DeSancho MT, Diuguid D, Griffin DO, Kahn SR (2021). American Society of Hematology 2021 guidelines on the use of anticoagulation for thromboprophylaxis in patients with COVID-19. Blood advances.

[10] Gordon-Cappitelli J, Choi MJ (2020). Prophylactic anticoagulation in adult patients with nephrotic syndrome. Clin J Am Soc Nephrol.

[11] Harza M, Ismail G, Mitroi G, Gherghiceanu M, Preda A, Mircescu G, Sinescu I (2013). Histological diagnosis and risk of renal vein thrombosis, and other thrombotic complications in primitive nephrotic syndrome. Romanian J Morphol Embryol.

[12] Porfidia A, Valeriani E, Pola R, Porreca E, Rutjes AW, Di Nisio M (2020). Venous thromboembolism in patients with COVID-19: systematic review and meta-analysis. Thromb Res.

[13] Singh B, Kaur P (2021). COVID-19 and acute mesenteric ischemia: a review of literature. Hematology, Transfusion and Cell Therapy.

[14] Enya T, Sugimoto K (2021). SARS-CoV-2 infection associated with the recurrence of nephrotic syndrome in a Japanese boy. Pediatr Nephrol.

[15] Harambat J, Allard L, Godron-Dubrasquet A (2021). Relapse rate of nephrotic syndrome in the time of COVID-19. Pediatr Nephrol.

[16] Alvarado A, Franceschi G, Resplandor E, Sumba J, Orta N (2021). COVID-19 associated with onset nephrotic syndrome in a pediatric patient: coincidence or related conditions?. Pediatr Nephrol.

[17] Melgosa M, Madrid A, Alvárez O, Lumbreras J, Nieto F, Parada E, Perez-Beltrán V (2020). SARS-CoV-2 infection in Spanish children with chronic kidney pathologies. Pediatr Nephrol.

[18] Doevelaar AA, Hölzer B, Seibert FS, Bauer F, Stervbo U, Rohn BJ, Zgoura P, Schenker P, Vonbrunn E, Amann K, Viebahn R (2020). Lessons for the clinical nephrologist: recurrence of nephrotic syndrome induced by SARS-CoV-2. Journal of nephrology.

[19] Cristoforo T, McKinley G, Ambrosio P. Saddle pulmonary embolism in a pediatric patient with nephrotic syndrome and recent COVID-19 pneumonia: a case report. Am J Emerg Med. 2021;16. 10.1016/j.ajem.2021.04.014 PMC8049848 33958247

[20] Han C, Duan C, Zhang S, Spiegel B, Shi H, Wang W, et al. Digestive symptoms in COVID-19 patients with mild disease severity: clinical presentation, stool viral RNA testing, and outcomes. Am J Gastroenterol. 2020;1. 10.14309/ajg.0000000000000664 PMC7172493 32301761

[21] Munyentwali H, Bouachi K, Audard V, Remy P, Lang P, Mojaat R, Deschênes G, Ronco PM, Plaisier EM, Dahan KY (2013). Rituximab is an efficient and safe treatment in adults with steroid-dependent minimal change disease. Kidney Int.

[22] Lavin M, Elder PT, O’Keeffe D, Enright H, Ryan E, Kelly A, El Hassadi E, McNicholl FP, Benson G, Le GN, Byrne M (2021). Vaccine-induced immune thrombotic thrombocytopenia (VITT)–a novel clinico-pathological entity with heterogeneous clinical presentations. Br J Haematol.

[23] Crous-Bou M, Harrington LB, Kabrhel C. Environmental and genetic risk factors associated with venous thromboembolism. InSeminars in thrombosis and hemostasis 2016 Nov (Vol. 42, no. 8, p. 808). NIH Public Access. 10.1055/s-0036-1592333 PMC5146955 27764878

